# A Novel Ferroptosis-Related lncRNAs Signature Predicts Clinical Prognosis and Is Associated With Immune Landscape in Pancreatic Cancer

**DOI:** 10.3389/fgene.2022.786689

**Published:** 2022-03-07

**Authors:** Haiqin Ping, Xingqing Jia, Hengning Ke

**Affiliations:** ^1^ Department of Infectious Disease, Hubei AIDS Clinical Training Center, Zhongnan Hospital of Wuhan University, Wuhan, China; ^2^ Jinan City People’s Hospital, Jinan, China; ^3^ Wuhan Research Center for Infectious Diseases and Cancer, Chinese Academy of Medical Sciences, Wuhan, China; ^4^ Cancer Research Institute, General Hospital, Ningxia Medical University, Yinchuan, China

**Keywords:** pancreatic cancer, ferroptosis, lncRNAs, clinical prognosis, immune landscape

## Abstract

Pancreatic cancer is one of the most lethal malignancies and currently therapies are severely lacking. In this study, we aimed to establish a novel ferroptosis-related lncRNAs signature to predict the prognosis of patients with pancreatic cancer and evaluate the predictive abilities of candidate lncRNAs. According to The Cancer Genome Atlas (TCGA) database, a total of 182 patients with pancreatic cancer were included in our study. Ferroptosis-related lncRNAs were screened by Pearson correlation analysis with 60 reported ferroptosis-related genes. Through univariate, least absolute shrinkage and selection operator (LASSO) regression and multivariate regression analyses, a novel signature based on five ferroptosis-related lncRNAs(*ZNF236-DT, CASC8, PAN3-AS1, SH3PXD2A-AS1, LINP1*) was constructed. Risk-related differentially expressed genes (DEGs) were subjected to enrichment analyses for Gene Ontology (GO), Kyoto Encyclopedia of Genes and Genomes (KEGG) pathway enrichment analysis. The results revealed that immune cell infiltration, immune-related functions and checkpoints were factors to affect prognoisis of pancreatic cancer. In summary, we identified the prognostic ferroptosis-related lncRNAs(*ZNF236-DT, CASC8, PAN3-AS1, SH3PXD2A-AS1, LINP1*) in pancreatic cancer and these lncRNAs may serve as therapeutic targets for pancreatic cancer.

## Introduction

Pancreatic cancer is one of the most lethal malignancies with a high mortality rate, and the incidence has steadily increased in the past few years. A family history of pancreatic cancer has been considered as an important risk factor for the disease (Raimondi et al., 2009), other risk factors include cigarette smoking, gender, advancing age, diabetes and so on. Early-stage pancreatic cancer is often asymptomatic. Symptoms usually occur after the surrounding tissues has been invaded or tumours metastasized to distant organs. As a result, the disease has already progressed to an advanced stage at the time of diagnosis (Gillen et al., 2010; Kamisawa et al., 2016). So far, surgery is considered the only potentially curative therapy for patients with pancreatic cancer. Unfortunately, many patients eventually experience recurrence after surgery and the impact of pancreatectomy on the quality of life and long-term outcomes is uncertain (Siegel et al., 2014). Therefore, identifying biomarkers for prognosis are of great clinical significance for patients.

Ferroptosis is a novel form of regulated cell death driven by iron-dependent lipid peroxidation (Xie et al., 2016a). Intracellular iron accumulation and lipid peroxidation are two central events in ferroptosis. Ferroptosis induced by small-molecule compounds or chemical drugs can suppress the growth of many types of tumors and improve the efficacy of chemotherapy, radiotherapy, or immunotherapy. In recent years, it has become a new strategy for treating many types of tumors including those from iron-rich tissues such as pancreas (Eling et al., 2015; Xie et al., 2016b). Research showed that the deletion of a system X_C_-subunit, SLC7A11, induced tumor-selective ferroptosis and inhibited pancreatic ductal adenocarcinoma growth (Badgley et al., 2020).

The long non-coding RNAs(lncRNAs) are a class of RNAs with a minimum length of about 200 nucleotides and with non-protein-coding ability (Guttman and Rinn, 2012). The function of lncRNAs is not fully understood. It may be involved in the regulation of genes by affecting translational regulation, histone modifications, or post-transcriptional processes (Huang et al., 2018). Studies have shown that lncRNAs play an important role in the regulation of cell cycle and cell differentiation in, and thus affect the development of tumors (Ramilowski et al., 2020). Research on lncRNAs has become a hot topic, but there are few studies on ferroptosis related lncRNA, especially in pancreatic cancer. In this article, we aimed to establish a novel ferroptosis-related lncRNAs (FRlncRNAs) signature to predict the prognosis of patients with pancreatic cancer and evaluate the predictive abilities of candidate lncRNAs. This study identified multiple ferroptosis-related lncRNAs as potential biomarkers for pancreatic cancer prognosis and further studies will improve current diagnosis, treatment, follow-up and prevention strategies of this disease.

## Materials and Methods

### Dataset

The public RNA sequencing of 182 patients and full clinical information were obtained from the GDC data portal using gdc-client (https://gdc-portal.nci.nih.gov/). The clinical characteristics including (age, gender, clinical grade and clinical stage) and survival overall survival (OS) information. Patients without survival information were excluded from further evaluation. In addition, the expression level of 60 ferroptosis-related genes (ACSL4, AKR1C1, AKR1C2, AKR1C3, ALOX15, ALOX5, ALOX12, ATP5MC3, CARS1, CBS, CD44, CHAC1, CISD1, CS, DPP4, FANCD2, GCLC, GCLM, GLS2, GPX4, GSS, HMGCR, HSPB1, CRYAB, LPCAT3, MT1G, NCOA4, PTGS2, RPL8, SAT1, SLC7A11, FDFT1, TFRC, TP53, EMC2, AIFM2, PHKG2, HSBP1, ACO1, FTH1, STEAP3, NFS1, ACSL3, ACACA, PEBP1, ZEB1, SQLE, FADS2, NFE2L2, KEAP1, NQO1, NOX1, ABCC1, SLC1A5, GOT1, G6PD, PGD, IREB2, HMOX1, ACSF2) were constructed from the dataset.

### Identification of Prognostic Ferroptosis-Related lncRNAs

Based on the lncRNA annotation file downloaded from the GENCODE (https://www.gencodegenes.org/human/) website and Ensemble IDs, we annotated 14,086 lncRNAs and 19,604 mRNAs according in the TCGA datdset. Pearson correlation analysis was used to evaluate the ferroptosis-related lncRNA. LncRNAs with *p* value <0.01 and an absolute Pearson correlation coeffcient≥ 0.4 or ≤0.4 were selected as ferroptosis-related lncRNAs. Then, the univariate cox regression analysis was used to screen out the prognostic lncRNAs(*p* < 0.05).

### Prognostic Score Risk Model Based on Independent Prognostic Ferroptosis-Related LncRNAs

The risk model was constructed through the Least Absolute Shrinkage and Selection operator (LASSO) and multivariate Cox regression analysis. To improve the accuracy of the statistical model, Lasso Cox regression analysis was carried out by using the R package “glmnet”. There are 10 ferroptosis-related lncRNAs were screened to construct the best prognostic model through the multivariate Cox regression analysis. The risk score was generated using the following formula:

risk score = ∑CoeflncRNAs × ExplncRNAs

All patients were divided into high-risk and low-risk groups according to the median cutoff of risk score.

### Survival Analysis

The Kaplan-Meier survival method was applied to evaluate the availability of the prognostic model and all the statistical analyses were conducted using R language (version 4.0). In addition, the area under the ROC curve (AUC) was calculated to evaluate the prognostic accuracy and sensitivity of the model. Survival analysis was also performed for the five crucial lncRNAs(*ZNF236-DT, CASC8, PAN3-AS1, SH3PXD2A-AS1, LINP1*) respectively in the model. We divided pancreatic cancer patients into different subgroups and compared whether there were differences in risk scores among the groups.

### Establishment a Nomogram

Nomogram is a tool that can personally calculate the survival rate of patients with specific tumors and has great value in clinical application (Park, 2018). So, we constructed a nomogram to help clinicians conveniently use our model to predict the overall survival at 1, 3, and 5 years of patients with pancreatic cancer. The nomogram is completed by use the R package “rms”, “Hmisc”, it includes risk score, age, gender, grade and stage.

### Functional Enrichment Analysis

We identified differentially expressed genes (DEGs) between low-risk and high-risk subgroups in the TCGA cohort in order to investigate potential biological functions and pathways between the two groups. All the DEGs in the study were performed for Gene Ontology (GO) and Kyoto Encyclopedia of Genes and Genomes (KEGG) pathway enrichment analysis by using the clusterProfile R package, which the results with *p* < 0.05 were considered significant.

### Evaluation of the Immune Landscape

The proportion of tumor-infiltrating immune cells between two risk groups were calculated by CIBERSORT, ESTIMATE, single-sample gene set enrichment analysis (ssGSEA) algorithms based on FRlncRNAs signature. The results were filtered with *p* value < 0.05.

Immune checkpoints are the immunosuppressive pathways that immune cells possess to regulate and control the persistence of the immune responses (Abril-Rodriguez and Ribas, 2017). The up-regulation of immune checkpoint protein is also one of the mechanisms by which tumor cells evade immune responses (Pardoll, 2012). In recent years, checkpoint blockade therapies have become a new type of cancer treatment. Thus, we investigated whether there were differences in the expression of immune checkpoint genes between the two risk groups, which aimed to investigate the potential role of FRlncRNAs signature in immune checkpoint blockade therapy.

Finally, we used a variety of currently acknowledged methods to analyze the relationship between risk and immune-cell characteristics, including XCELL, TIMER, QUANTISEQ, MCPOUNTER, EPIC, CIBERSORT-ABS and CIBERSORT. The relationship between the risk score and the immune infiltrated cells was performed by Spearmen correlation analysis. The correlation coefficient is bounded by 0 and the results were shown in a lollipop diagram.

### Cell Culture and Knockdown of SH3PXD2A-AS1 and LINP1

Human pancreatic cancer cells PANC-1 were used in our experiment. Cells were cultured in high-glucose Dulbecco’s Modified Eagle’s Medium (supplemented with 10% fetal bovine serum and 1% penicillin/streptomycin) at 37°C and 5% CO2 in a cell incubator. The siRNA against SH3PXD2A-AS1 (siSH3PXD2A-AS1), the LINP1(siLINP1) and the control siRNA (siNC) were purchased from RiboBio Corporation (Guangzhou, China). The siRNAs transfection were performed using Lipofectamine 2000 transfection reagent. The sequence of siLINP1 and siSH3PXD2A-AS1 were showed below:

siLINP1

5′-CCA​ACT​GCG​GGA​CTT​CAG​A dTdT-3′(sense), 5′-TCT​GAA​GTC​CCG​CAG​TTG​G dTdT- 3′(antisense).

siSH3PXD2A-AS1

5′-CCC​TAA​GGA​CAG​AAT​GCA​A dTdT-3′(sense), 5′- TTG​CAT​TCT​GTC​CTT​AGG​G dTdT- 3′(antisense).

### Wound Healing Assay

PANC-1 cells were grown in six-well plates until they formed a tight cell monolayer. A 200ul sterile tip was used to form a wound in the middle of the cell. Cells were then cultured in medium supplemented with 2% FBS. The width of wounds were photographed using a microscope at 0 and 24 h. The percentage of wound healing was generated using the following formula: average of ([gap area: 0 h]–[gap area: 24 h])/[gap area: 0 h]).

### Transwell Assay

Firstly, PANC-1 cells in 200 µl of serum-free DMEM were incubated to the upper chamber which is coated with Matrigel, 500 µl of complete medium was placed in the lower chamber. The upper chambers containing non-invasive cells were removed with cottons swabs after 48 h. Then, the lower chamber was fixed with 4% paraformaldehyde and stained in crystal violet. The chambers were observed under microscope.

### Cell Proliferation Assay

For Cell Counting Kit-8 (CCK-8) assays, cells transfected for 48 h were collected and seeded into a 96-well plate. According to the instructions, the optical density (OD) values were measured at a 450-nm wavelength after the cells were incubated for 2 h at 37°C.

## Results

### Identification of Ferroptosis-Related lncRNAs in Pancreatic Cancer

**FIGURE 1:**
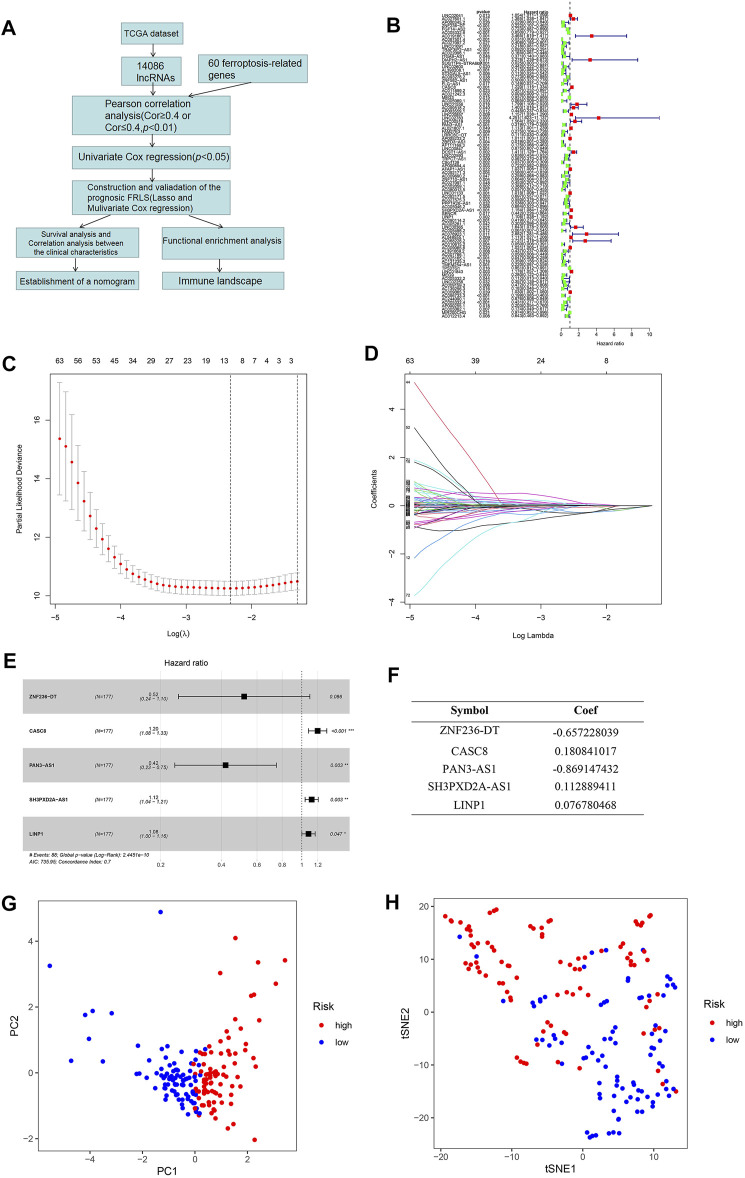
**(A)** The flow diagram of the research process. **(B)** Forest plots revealed prognosis-related ferroptosis-related lncRNAs based on the results of univariate Cox regression. **(C,D)** The least absolute shrinkage and selection operator (LASSO) regression was performed with the minimum criteria. An optimal log λ value is indicated by the vertical black line in the plot. **(E)** The forest plot of the multivariate Cox regression analysis. **(F)** The coefficients of five selected ferroptosis-related lncRNAs measured by the multivariate Cox. **(G)** PCA plot showing the distribution of the established FRLS expression in different risk groups. **(H)** t-SNE plot showing the distribution of the patients in different risk groups.

There are a total of 182 patients from the TCGA dataset who were identified and included in this study. 14,086 lncRNAs in the TCGA datasets were obtained by matching the lnRNA annotation with the ENSEMBL ID downloaded from the “GENCODE” website. 60 ferroptosis-related genes were collected from the published literature for subsequent analysis. LncRNAs with *p* value < 0.01 and an absolute Pearson correlation coeffcient ≥ 0.4 or ≤ 0.4 were selected as ferroptosis-related lncRNAs. Among 246 ferroptosis-related lncRNAs, 89 lncRNAs were identified as prognostic ferroptosis-related lncRNAs through the univariate cox regression analysis (*p* < 0.05). The research process is shown in Figure 1A and the forest map of these 89 lncRNAs is shown in Figure 1B.

### Construction of the Ferroptosis-Related lncRNAs Signature (FRLS) in the TCGA Dataset

To build the FRLS for the patients of pancreatic cancer in TCGA dataset, the above 89 prognostic ferroptosis-related lncRNAs were incorporated into the least absolute shrinkage and selection operator (LASSO) regression firstly (Figures 1C,D). 10 ferroptosis-related lncRNAs including *ZNF236-DT, AC005332.6, AC019186.1, AC087501.4, CASC8, PAN3-AS1, LINC01133, SH3PXD2A-AS1, LINP1, AC090114.2* were generated. To further optimize the results, we performed the multivariate Cox regression analysis to construct a prognostic model for OS using the expression level of the 10 ferroptosis-related lncRNAs. An optimal 5 lncRNAs (*ZNF236-DT, CASC8, PAN3-AS1, SH3PXD2A-AS1, LINP1*) signature and coefficient of each were identified (Figures 1E,F). The forest plot shows that *ZNF236-DT* and *PAN3-AS1* are protective factors with HR (Hazard Ratio) < 1, while *CASC8, SH3PXD2A-AS1* and *LINP1* are risk factors with HR > 1 (Figure 1E). The patients were further divided into high-risk group (*n* = 88) and low-risk group (*n* = 89). The groups were generated by the formula mentioned in section “*Materials and Methods*”. Principal Component Analyses (PCA) showed a significant distribution difference between high- and low-risk subgroups (Figures 1G,H).

**FIGURE 2:**
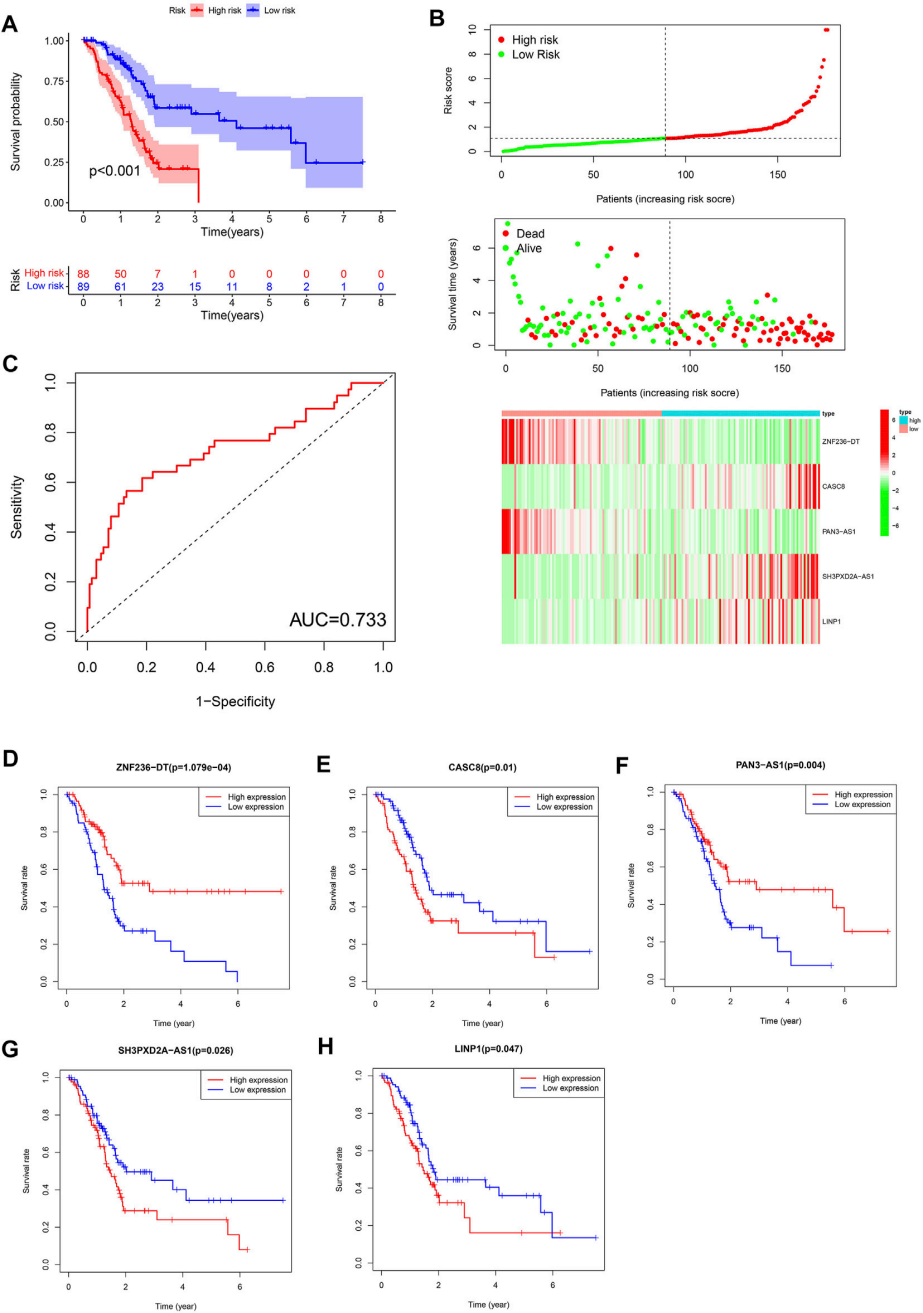
**(A)** Kaplan-Meier curves for the overall survival of patients in the high- and low-risk groups in the TCGA cohort. **(B)** Distributions of risk scores, survival status and expression of five ferroptosis-related lncRNAs in the TCGA dataset. **(C)** The receiver operating characteristic (ROC) curve analyses of the prognostic FRLS in the TCGA cohorts. **(D–H)** Kaplan-Meier curves analyzed on the correlation between the expression of the five crucial lncRNAs and the prognosis of pancreatic cancer.

The Kaplan-Meier survival analysis showed that the high-risk group (*n* = 88) had a lower probability of survival and a shorter overall survival (OS) time than patients in the low-risk group (*n* = 89) (Figure 2A). The area under ROC curve (AUC) reached 0.733, which demonstrated that the FRLS could be used to predict OS in the TCGA cohort (Figure 2C). Risk score, survival status and heatmap are plotted in Figure 2B. Then, the 5 crucial lncRNAs(*ZNF236-DT, CASC8, PAN3-AS1, SH3PXD2A-AS1, LINP1*) of the FRLS were evaluated by survival analysis. The Kaplan–Meier survival curves confirmed the forest plot shown in Figure 1E, which means higher expressions of *ZNF236-DT, PAN3-AS1* and lower expressions of *CASC8, SH3PXD2A-AS1* and *LINP1* were associated with better overall survival (OS) (Figures 2D–H).

### Correlation Analysis Between the FRLS and Clinicopathological Features

**FIGURE 3:**
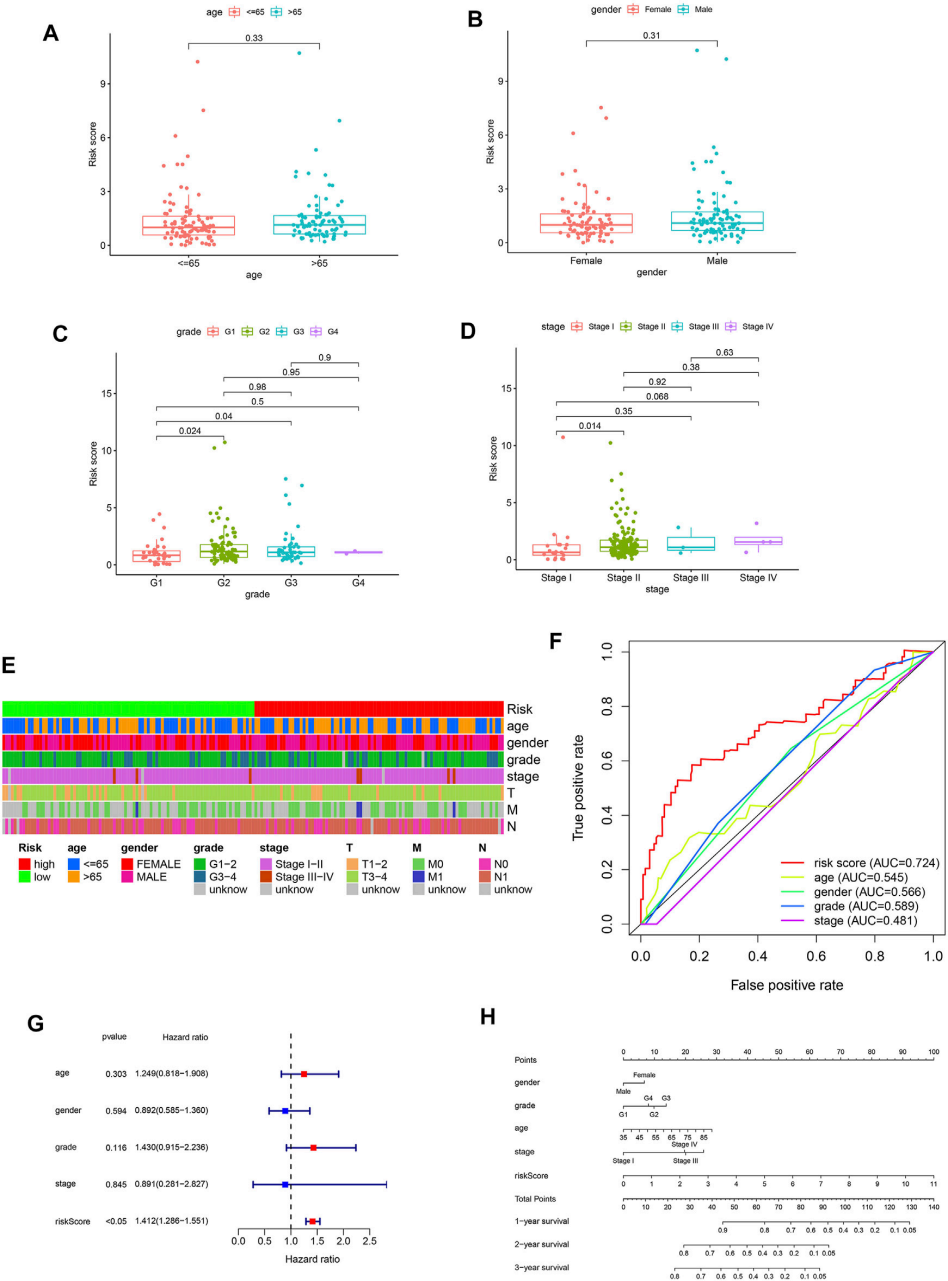
**(A–D)** Different levels of risk scores in pantients with pancreatic cancer stratified by age,gender, grade and stage. **(E)** A strip chart of the associations between risk score and clinicopathological features in the TCGA dataset. **(F)** The receiver operating characteristic (ROC) curves for the risk score, age, gender, grade and stage in the TCGA dataset. **(G)** Results of the univariate Cox regression analysis regarding OS in the TCGA cohort. Risk score was the only independent prognostic parameter. **(H)** Nomogram based on risk score, age, gender, grade and stage.

To further assess the prognostic efficacy of FRLS, we divided patients into various subgroups based on clinicopathological features and compared the levels of risk score between different groups. No risk score differences were observed between patients satisfied by gender and age (age was divided by 65 years old) (Figures 3A,B). But the results suggested that patients with the clinicopathological characteristics of grade 2 and 3, stage II have higher levels of risk score compared with other corresponding subgroups (Figures 3C,D). Thus, FRLS may have certain predictive value for clinicopathological features. The heatmap in Figure 3E further shows the distribution of clinicopathological characteristics and the risk groups.

Afterward, we have used univariate and multivariate Cox analyses to identify independent prognostic factors indicator. The results of univariate Cox and multivariate Cox regression analysis showed that only the risk score was significantly associated with OS in TCGA dataset (univariate Cox analyses: HR = 1.412, 95% CI = 1.286–1.551, *p* < 0.05) (Figure 3G). As shown in Figure 3F, the AUC value of the risk score was 0.724, higher than the AUC values of other clinicopathological factors. These results confirmed the FRLS is an independent and reliable prognostic indicators for pancreatic cancer.

In summary, we constructed a nomogram using the risk score (based on FRLS) and clinicopathological features, including age, gender, WHO grade and stage in the TCGA dataset (Figure 3H).

### Functional Enrichment Analysis

**FIGURE 4:**
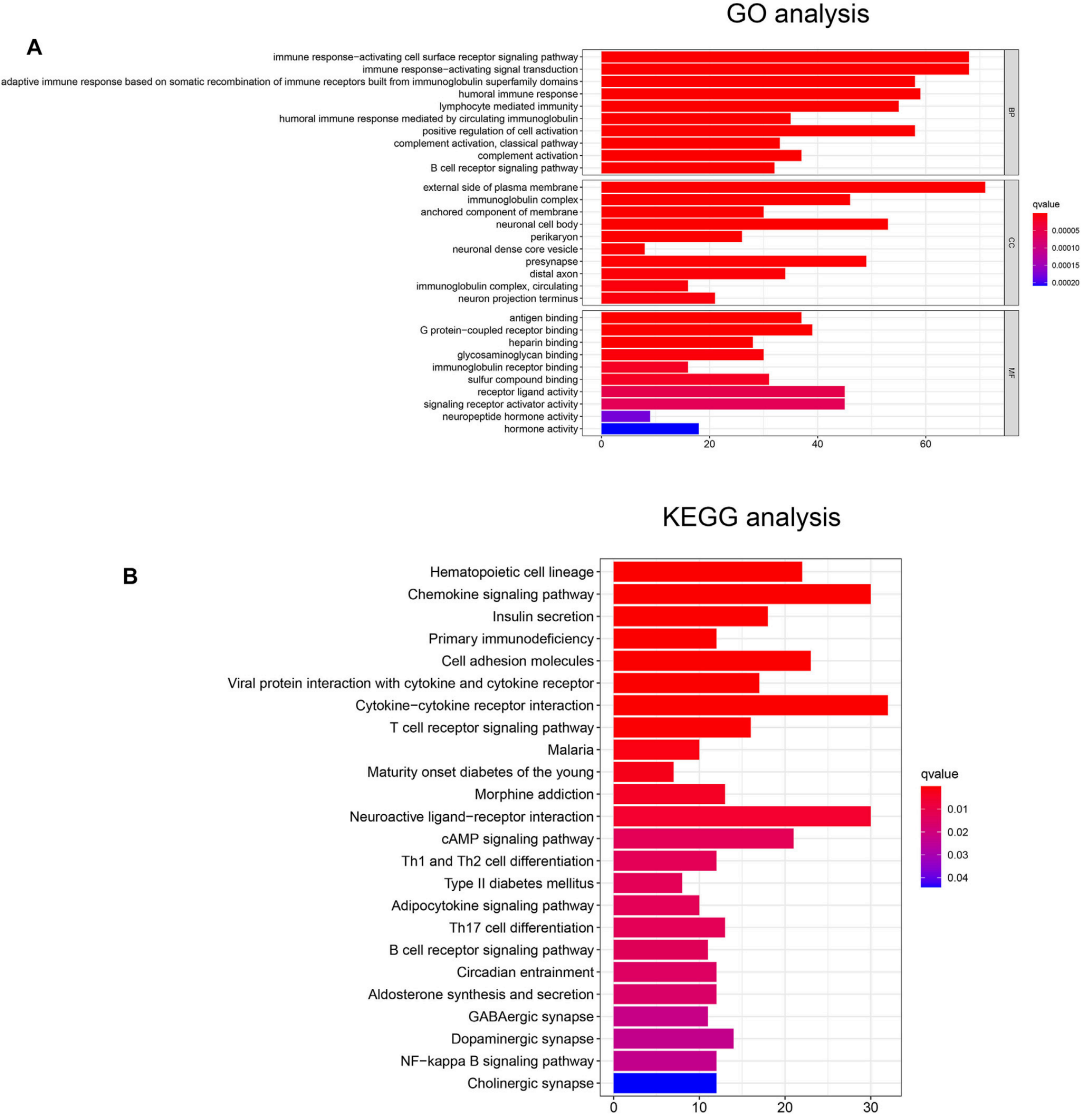
**(A)** The GO analysis of DEGs between the high- and low-risk groups. **(B)** The KEGG analysis of DEGs between the high- and low-risk groups.

To investigate potential biological functions and pathways between the two risk subgroups, we performed functional enrichment analysis of the differentially expressed genes (DEGs) between the two groups. There are 973 DEGs [|log2 (fold change)| > 1 and *p* < 0.05] between the low- and high-risk subgroups. Interestingly, the GO analysis revealed that the DEGs were highly enriched in several immune-related biological processes, including immune response-activating cell surface receptor signaling pathway, immune response-activating signal transduction, humoral immune response, lymphocyte mediated immunity, complement activation, B cell receptor signaling pathway (Figure 4A). Similarly, the KEGG analysis also showed significant enrichment of DEGs in immune-related pathways, for instance, cytokine-cytokine receptor interaction, chemokine signaling pathway, T cell receptor signaling pathway, B cell receptor signaling pathway (Figure 4B).

### Correlation of the Prognostic FRLS With the Immune Landscape

**FIGURE 5:**
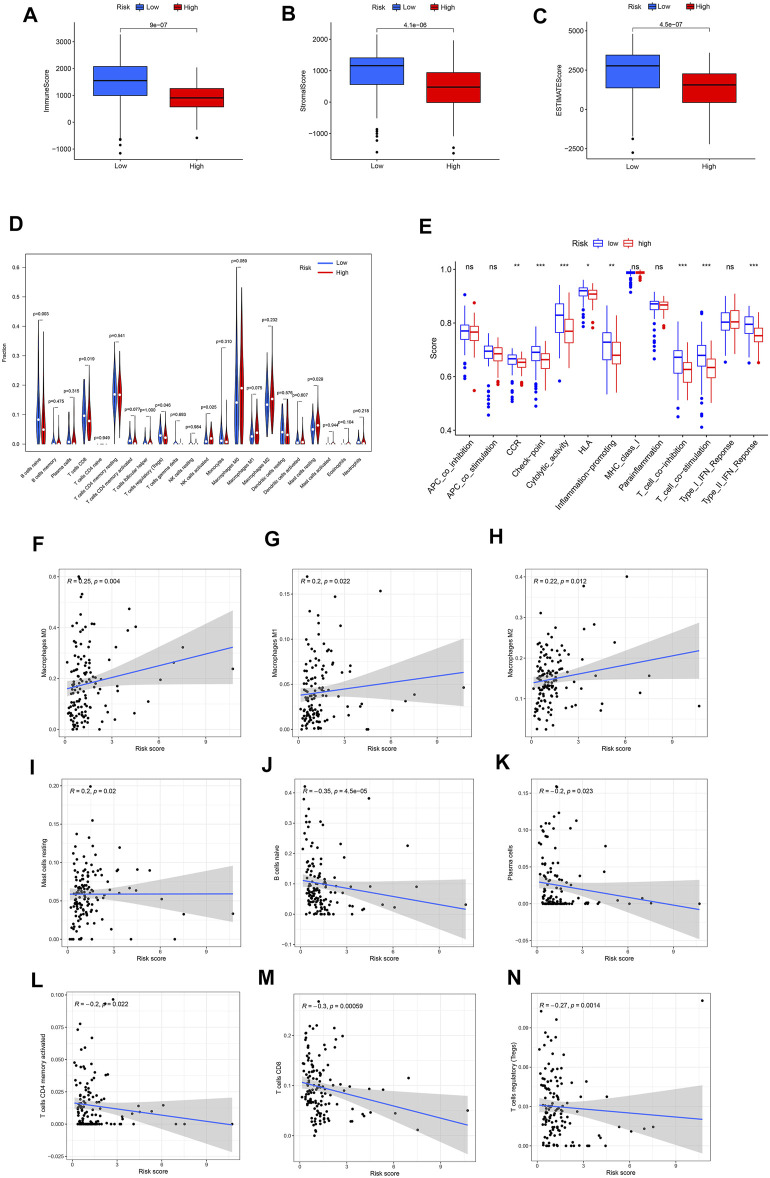
**(A–C)** Different expression of immune score, stromal score and ESTIMATE score in the high- and low-risk groups. **(D)** Violin plot visualizing the differentially infiltrated immune cells. **(E)** The ssGSEA scores of 13 immune-related functions between different risk groups in the TCGA cohort. **(F–N)** Relationships between the FRLS and infiltration abundances of nine types of immune cells. The correlation was performed by Pearson correlation analysis. **(F)** Macrophages M0; **(G)** Macrophages M1; **(H)** Macrophages M2; **(I)** Mast cells resting; **(J)** B cells naive; **(K)** Plasma cells; **(L)** T cells CD4 memory activated; **(M)** T cells CD8; **(N)** T cells regulatory. Treg, regulatory T cell; APC,antigen presenting cell; CCR, cytokine-cytokine receptor; HLA, human leukocyte antigen; MHC, major histocompatibility complex; IFN, immune interferon; ns, not significant; ***p < 0.001; **p < 0.01; *p < 0.05.

Since the KEGG and GO analysis suggested that DEGs were enriched in immune-related functions and pathways, we further investigated the immune landscape in TCGA dataset. The ESTIMATE algorithm showed that the immune score, stromal score and ESTIMATE score were all higher in the low-risk group than in the high-risk group (Figures 5A–C). These results also suggest that tumor purity was lower in the low-risk group than in the high-risk group.

Moreover, the CIBERSORT algorithm was used to analyze the 22 different immune cell types among the two risk group. As shown in Figures 5B,D cell naive, T cell CD8 and T cells regulatory (Tregs) were up-regulated in the low risk subgroup of the TCGA cohort, while NK cells activated and mast cell resting were significantly down-regulated (*p* < 0.05). Next,we further analyzed the correlation between FRLS and immune cells. The results indicated that this signature was most significantly positive correlation with immune infiltration of Macrophages M0(Cor = 0.25, *p* = 0.004), Macrophages M1(Cor = 0.2, *p* = 0.022), Macrophages M2(Cor = 0.22, *p* = 0.012) and Mast cells resting (Cor = 0.2, *p* = 0.02), but negatively correlated with B cells naive (Cor = −0.35, *p* = 4.5e-05), Plasma cells (Cor = −0.2, *p* = 0.023), T cells CD4 memory activated (Cor = −0.2, *p* = 0.022), T cells CD8 (Cor = −0.3, *p* = 0.00059) and T cells regulatory (Tregs, Cor = −0.27, *p* = 0.0014) (Figures 5F–N). These findings again confirmed that this FRlncRNAs signature was associated to immune cell infiltration in pancreatic cancer.

**FIGURE 6:**
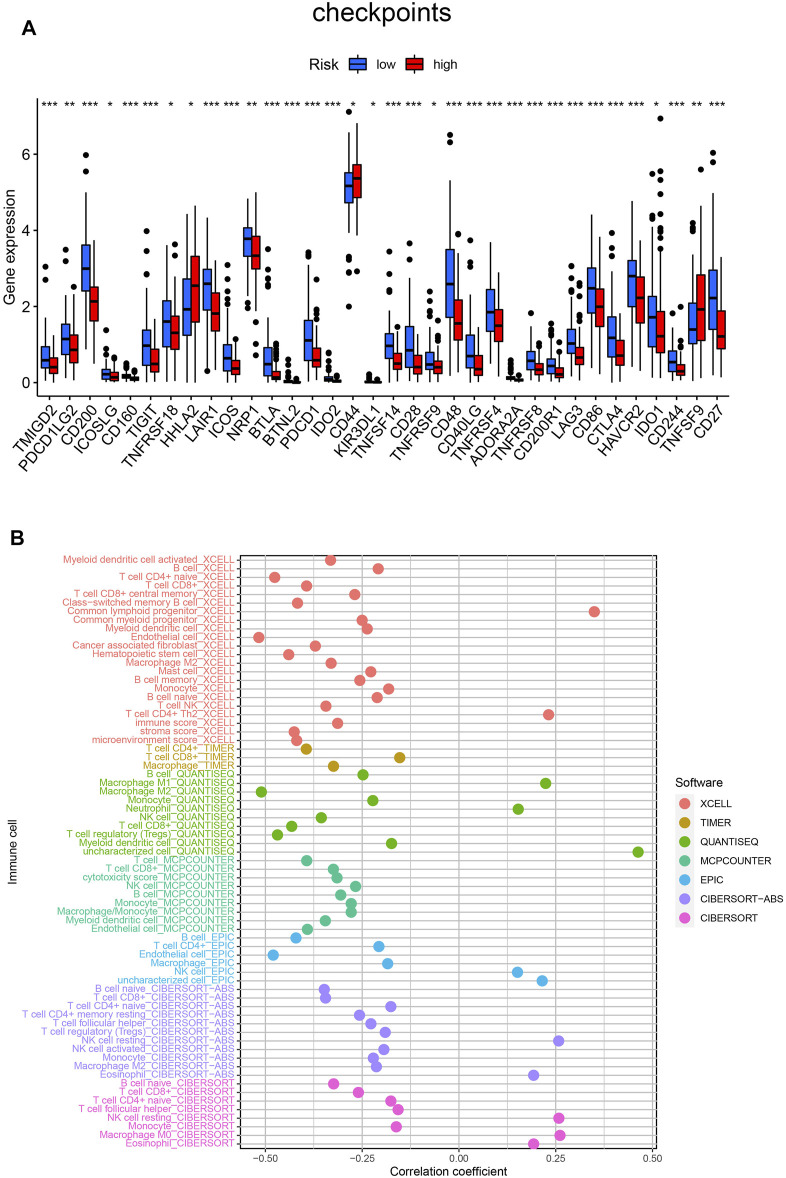
**(A)** Differences expression of thirty-four immune checkpoints in the two risk groups. **(B)** Estimation of tumor-infiltrating immune cells based on XCELL, TIMER, QUANTISEQ, MCPOUNTER, EPIC, CIBERSORT-ABS and CIBERSORT algorithms. ***p < 0.001; **p < 0.01; *p < 0.05.

By using the ssGSEA analysis, we quantified the enrichment scores of immune-related functions in the two risk groups. The results showed that the high-risk group was significantly correlated with most immune-related functions, such as CCR, Check-point, Cytolytic-activity, HLA, Inflammation-promoting, T cell co-inhibition, T cell co-stimulation and Type II IFN Response (Figure 5E). Moreover, the expression of immune checkpoint molecules also differed significantly between the two risk groups, such as HHLA2, CD44 and TNFSF9 in the high-risk group were higher than those in the low-risk group, while other molecules were higher in the low-risk group (Figure 6A).

Finally, detailed Spearman correlation analysis was conducted. The risk score was positively correlated with common lymphoid progenitor, CD4^+^ T cells, Macrophage M1, Macrophage M0, Neutrophil, NK cells, Eosinophil and Monocyte. It was inversely associated with most other tumor-infiltrating immune cells (Figure 6B).

### Knockdown of Both LINP1 and SH3PXD2A-AS1 Suppressed Pancreatic Cancer Cell Proliferation, Invasion and Migration

Wound healing assay, transwell assay and cell proliferation assay were performed in order to investigate the effect of LINP1 and SH3PXD2A-AS1 on invasion, migration, and proliferation of pancreatic cancer cells. As shown in Supplementary Figures S3A,B, LINP1 and SH3PXD2A-AS1 knockdown in PANC-1 inhibited the wound closure compared to the control cells. Similarly, LINP1 and SH3PXD2A-AS1 knockdown resulted in the decreased of invasive cells and cell viability (Supplementary Figures S3C–E) compared to the control group. These results indicated that downregulation of LINP1 and SH3PXD2A-AS1 can inhibit the proliferation, invasion and migration of pancreatic cancer cells.

## Discussion

In this study, a total of 182 patients with pancreatic cancer from the TCGA dataset were included to exploit the prognostic significance of ferrotosis-related lncRNAs. Firstly, we identified 89 prognostic ferroptosis-related lncRNAs through the univariate Cox regression analysis. Under the LASSO regression and multivariate Cox regression analysis, five lncRNAs(*ZNF236-DT, CASC8, PAN3-AS1, SH3PXD2A-AS1, LINP1*) of them were selected to establish the novel prognostic FRLS for predicting the OS of patients with pancreatic cancer. Among the five lncRNAs, *ZNF236-DT* and *PAN3-AS1* are protective factors, while *CASC8*, *SH3PXD2A-AS1* and *LINP1* are risk factors. Secondly, based on the median risk score, patients were divided into the low- and high-risk subgroups and the high-risk group had worse clinical outcomes than patients in the low-risk group. Functional enrichment analysis showed that the DEGs between the two subgroups were highly enriched in several immune-related biological processes and pathways. Finally, we compared the immune and stromal scores, the infiltration of different immune cells and expression levels of immune checkpoint molecules. These results confirmed differences in the immune landscape between the two risk subgroups.

Pancreatic cancer is a malignant tumor with a high mortality rate. So far, the underlying mechanism of pancreatic cancer is not completely understood. A number of studies have revealed the functions and regulatory roles of lncRNAs in pancreatic cancer behaviors. Hisashi Yoshimura revealed that *H19* has important roles in pancreatic cancer metastasis, and that inhibition of *H19* represents a novel candidate for pancreatic cancer therapy (Yoshimura et al., 2018). *SNHG15* could act as an oncogene in pancreatic cancer, further research found that *SNHG15* knockdown inhibited proliferative capacities and suppressed apoptotic rate of pancreatic cancer cells *in vitro*, and impaired *in vivo* tumorigenicity (Ma et al., 2017). In addition, the correlation between immune-related lncRNAs and pancreatic cancer has also been reported in some studies. Zhang et al. (2021) reported that 3 immune-related lncRNA pairs (AC244035.1_vs._AC063926.1, AC066612.1_vs._AC090124.1 and AC244035.1_vs._LINC01885) exhibit effective prognostic prediction performance in pancreatic cancer. However, there are few studies about ferroptosis-related lncRNAs, especially in pancreatic cancer.

In ferroptotic cells, cytological changes mainly include increased mitochondrial membrane density, decrease or disappearance of mitochondrial crest (Yagoda et al., 2007). Ferroptosis is a novel method for the destruction of cancer cells and it can be triggered by some exogenic small molecules, e.g., erastin, Ras-selective lethal small molecule 3, certain clinical drugs and even nano ferroptosis inducers. Notably, ferroptosis has also been shown to be associated with tumor immunotherapy (Wang et al., 2019a). Yu et al. (2021) constructed six ferroptosis-related gene signature prognostic model and this model was proven to be stable and effective in predicting the prognosis of pancreatic cancer.

lncRNAs are part of the noncoding RNAs family and several new studies have suggested that lncRNAs play an important role in ferroptosis of tumors. For instance, long noncoding RNA *LINC00336* was found to inhibit ferroptosis in lung cancer by functioning as a competing endogenous RNA via sponging *miR-6852* (Wang et al., 2019b). In addition, Mao et al. (2018) found that lncRNA *P53RRA* can bind to Ras GTPase-activating protein-binding protein 1 (*G3BP1*) and promote ferroptosis in breast cancer and lung cancer. Among the established 5 ferroptosis-related lncRNAs in this study, three have been found to be associated with tumor development. Silencing *CASC8* inhibited the proliferation, migration, and invasion of non-small cell lung cancer cells and promoted their sensitivity to osimertinib (Jiang et al., 2021). *LINP1* may be involved in the regulation of cell proliferation, cell adhesion and cell cycle-related biological processes in early stage pancreatic ductal adenocarcinoma (Shang et al., 2020). Consistent with the findings of A-Y Chen (Chen et al., 2020), we found that the proliferation, invasion, and migration ability of Pancreatic cancer cells decreased remarkably in LINP1 knockdown group, compared to the si-NC group. These results suggested that LINP1 is important in cancer cell proliferation and invasin. Mechanism studies have found that *SH3PXD2A-AS1* can directly interact with *p53* protein and regulate p53-mediated gene transcription in colorectal cancer, and eventually lead to increased cell proliferation, angiogenesis, and metastasis (Hou et al., 2021). Whatsmore, it has been reported that ferroptosis-related lncRNAs were prognostic risk factors in different types of tumors. Three ferroptosis-related lncRNAs (DUXAP8, LINC02609, and LUCAT1) (Xing et al., 2021) were reported to significantly correlate with the overall survival and clinicopathological features of kidney renal clear cell carcinoma. In another study (Fei et al., 2021), a ferroptosis-related lncRNA prognostic signature (FLPS), which included six ferroptosis-related lncRNAs, provided a new strategy for the prediction of prognosis in lung adenocarcinoma.

The DEGs between the two subgroups of the FRLS were highly enriched in several immune-related biological processes and pathways and the immune score, stromal score were significantly different between the two groups. B cells, CD8^+^ T cells, Tregs were significantly enriched in the low risk group, NK cells and mast cells were enriched in the high risk group. Among these types of cell, mast cells, B cells and Tregs are antigen presenting cell (APCs) that are capable of presenting processed antigens to T cells and activating the immune response. Besides, it is interesting that the immune-related functions also differed significantly between the two risk groups. These functions are related to the development and treatment of tumors. For instance, Hiraoka et al. (2020) found that the higher expression of HLA-I, HLA-E and HLA-G on pancreatic ductal adenocarcinoma cells is an unfavorable prognosticator. High cytolytic activity was confirmed to associate with increased expression of genes involved in multiple immune checkpoints (with the notable exception of PD-L1) in pancreatic cancer (Balli et al., 2017). Therefore, we assume that ferroptosis is closely related to the immune landscape of microenvironment in pancreatic cancer. However, the potential molecular mechanisms remain to be explored by in-depth experimental researches.

In addition, further analysis found that the low-risk group exhibited higher expression levels of immune checkpoints. Among these immune checkpoints, therapy that targets programmed death 1 or programmed death 1 ligand 1 (*PD-1/PD-L1*) has been rapidly developing as oncotherapy for various carcinomas, including pancreatic cancer. Whatsmore, *CD200* has been shown to promote immunosuppression in the pancreatic tumor microenvironment and targeting *CD200* may enhance activity of checkpoint immunotherapy (Choueiry et al., 2020). Although immunotherapies such as checkpoint inhibition monotherapy have yet to demonstrate efficacy, a growing body of evidence suggests that combination regimens including chemotherapy could unlock immunotherapy in pancreatic cancer. Our analysis offered the possibility of several potential checkpoint targets for pancreatic cancer, but the mechanism and efficacy remained to be validated in the future.

However, there are several limitations in our study. Firstly, our study is only based on the TCGA public database and the sample size was relatively small. Secondly, our study lacks a validation cohort set and prospective, multicenter, real-world data to be further verified.

## Conclusion

The present study implicates that several ferroptosis-related lncRNAs(*ZNF236-DT, CASC8, PAN3-AS1, SH3PXD2A-AS1, LINP1*) may serve as independent prognostic biomarkers for pancreatic cancer. And this research provides a theoretical basis for therapeutic targets.

## Data Availability

Publicly available datasets were analyzed in this study. This data can be found here: https://portal.gdc.cancer.gov/.

## Supplementary Material

Supplementary Figure S1
**(A)** The characteristics of the external validation cohorts in GEO and ICGC. **(B)** The validation results in the Canadian Pancreatic Cancer dataset of ICGC

Supplementary Figure S2 The results of internal validation in TCGA database. **(A,B)** The Kaplan-Meier curves for survival in the training cohort and validation cohort. **(C–F)** The distribution plots of the risk score and survival status in the training cohort and validation cohort. **(G–H)** The ROC curve analyses of the prognostic FRLS in the training cohort and validation cohort.

Supplementary Figure S3
**(A,B)** Wound healing assay was performed to measure the effect of LINP1 and SH3PXD2A-AS1 downregulation on PANC-1 cell migration. **(C,D)** Transwell assay was performed to measure the effect of LINP1 and SH3PXD2A-AS1 downregulation on PANC-1 cell invasion. **(E)** Cell proliferation assay was performed to measure the effect of LINP1 and SH3PXD2A-AS1 downregulation on PANC-1 cell proliferation. ****P* < 0.001; ***P* < 0.01; **P* < 0.05.
