# Supplementary figures and images for: A Novel Ferroptosis-Related lncRNAs Signature Predicts Clinical Prognosis and Is Associated With Immune Landscape in Pancreatic Cancer
Source: Front Genet. 2022 Mar 7;13:786689. doi: 10.3389/fgene.2022.786689 (PMC8940287; doi:10.3389/fgene.2022.786689)

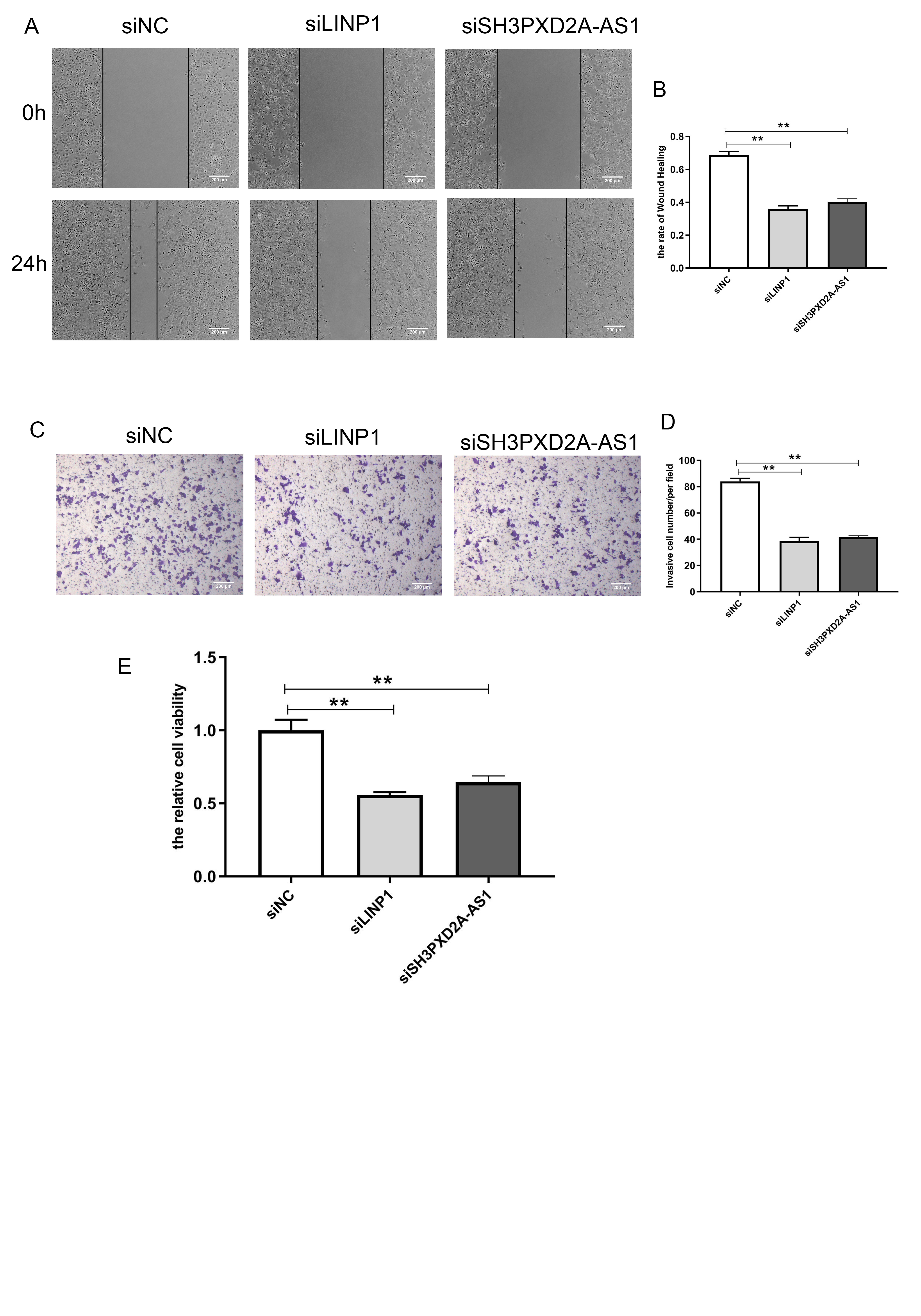

Supplement: Supplementary file 1 [file Image3.JPEG]

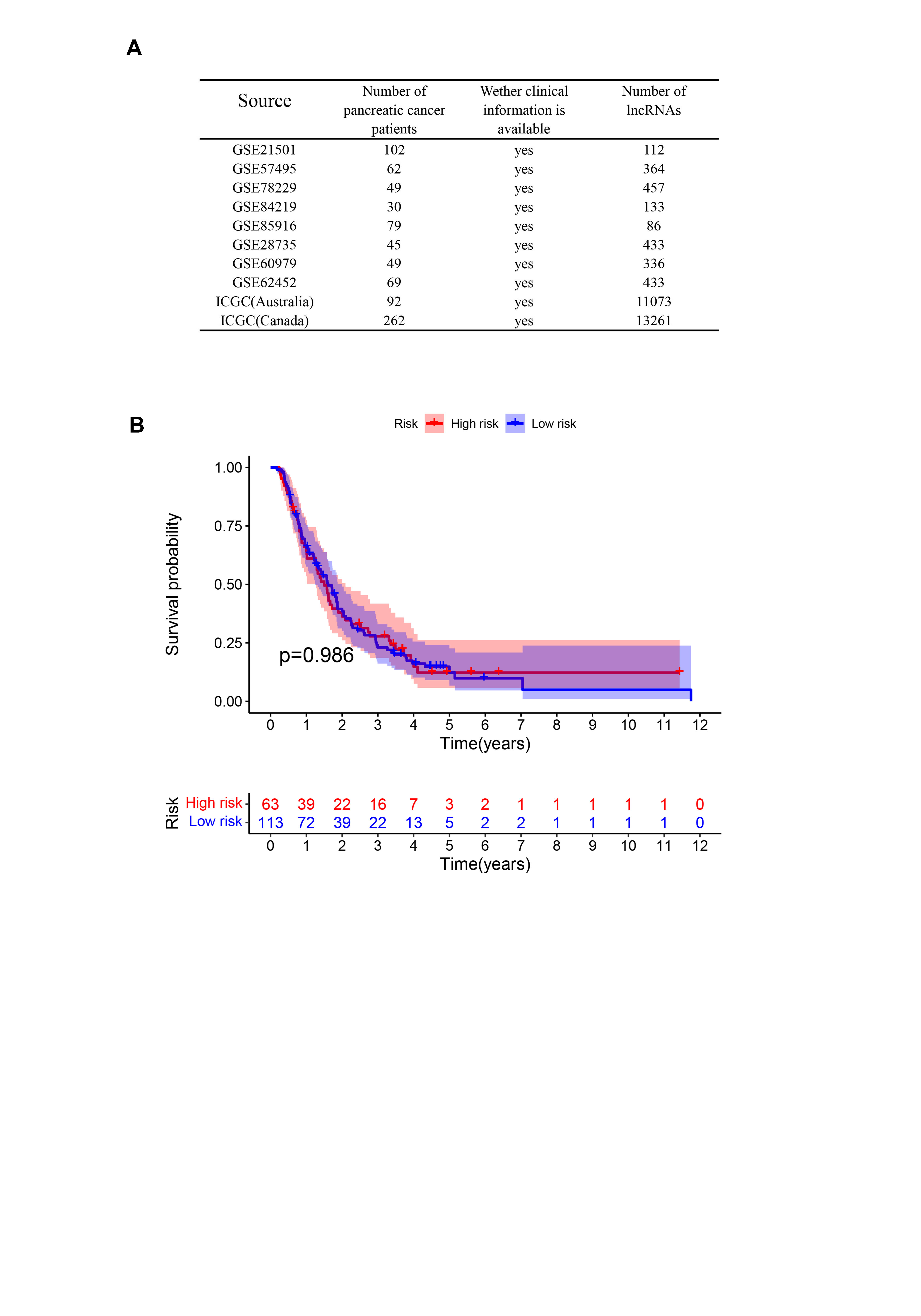

Supplement: Supplementary file 2 [file Image1.JPEG]

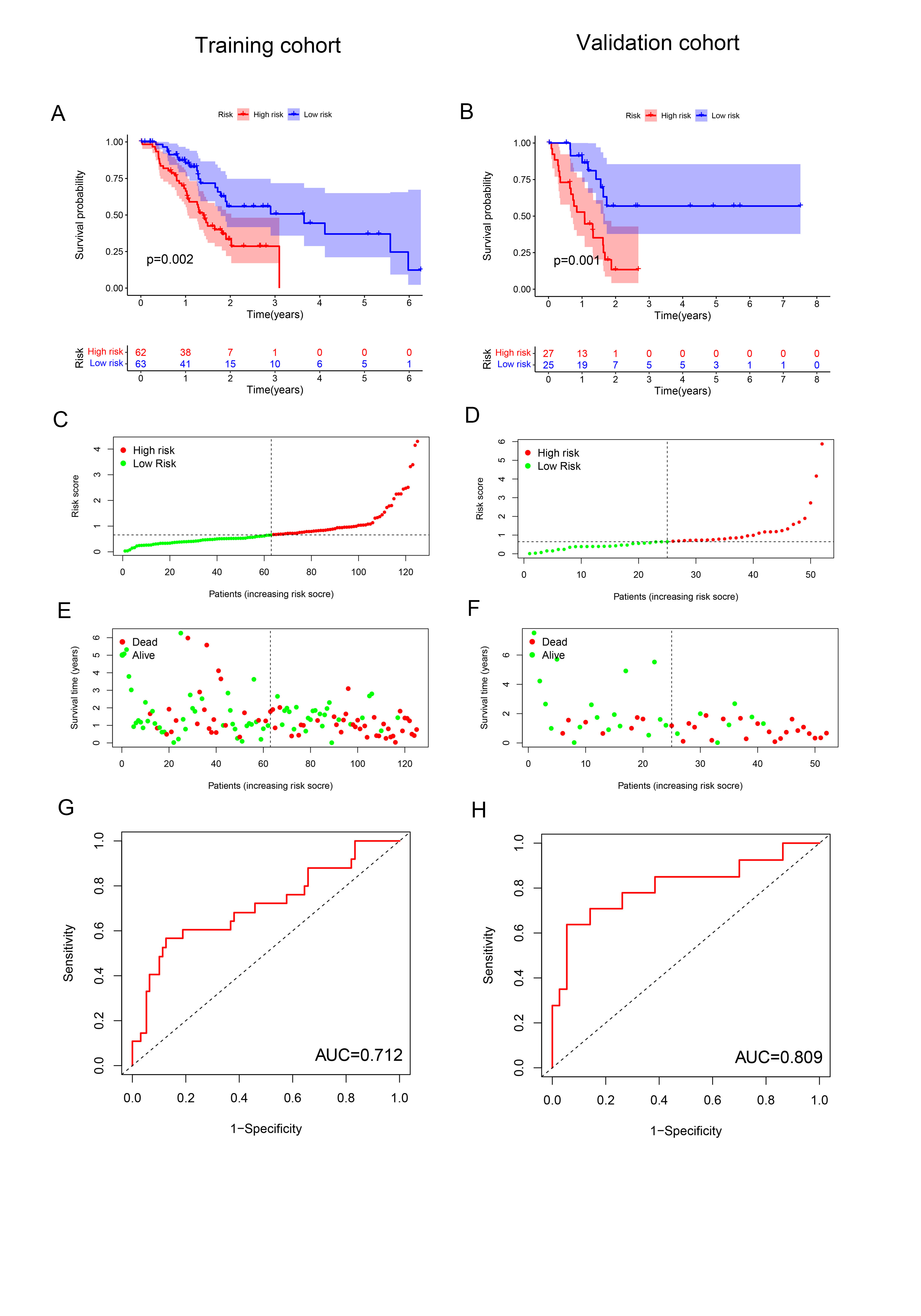

Supplement: Supplementary file 3 [file Image2.JPEG]
